# Supplementary material for: Identification of photoperiod-induced specific miRNAs in the adrenal glands of Sunite sheep (Ovis aries)
Source: Front Vet Sci. 2022 Jul 22;9:888207. doi: 10.3389/fvets.2022.888207 (PMC9354845; doi:10.3389/fvets.2022.888207)
Supplement: Supplementary file 1 [file Data_Sheet_1.ZIP › Supplementary file/miRDeep2 file.zip!/miRDeep2 file/Novel_Structure/SP42A2/Novel_362.pdf]

Provisional ID	:	NC_019458.2_321
Score total	:	2.6
Score for star read(s)	:	-1.3
Score for read counts	:	0
Score for mfe	:	2.3
Score for randfold	:	1.6
Score for cons. seed	:	
Total read count	:	81
Mature read count	:	81
Loop read count	:	0
Star read count	:	0

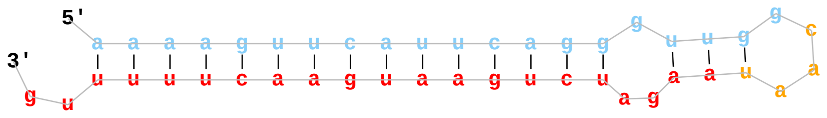

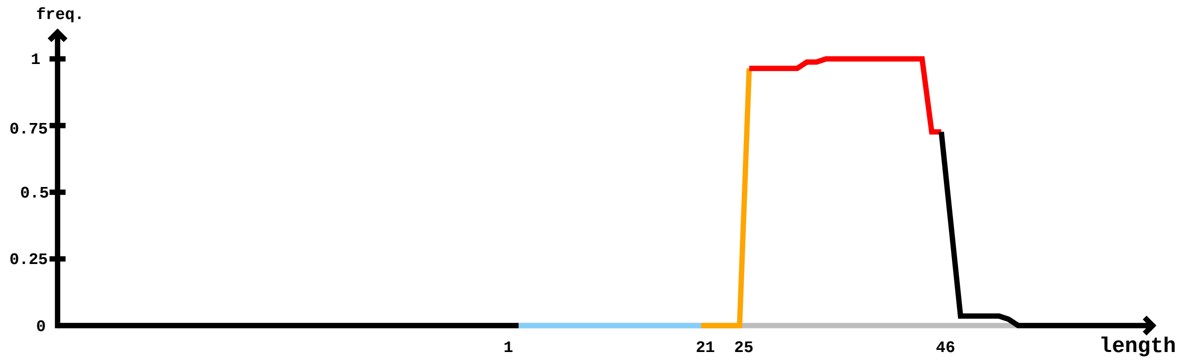

Star Mature

```
5'- uguguguauacauauguaugugugu auauauguauaggguuggccaaaaaguucauucaggguuggcaauaagaucugaaugaacuuuuuggccaauuauacacacacacac   -3'    exp
    (((((((((((((((((((....))))))))))....((((((((((((((((((((((((.(((....)))..))))))))))))))))))))))))))))))))).....    reads    mm    sample
    ......................................................................aaAaucugaaugaacuuuu.......................    23    1    seq
    ......................................................................aaAaucugaaugaacuuuuug.....................    57    1    seq
    ......................................................................aagaucugaaugaacuuuuugg....................    1    0    seq
    ...........................................................................ugaaugaacuuuuuggccaauu...............    2    0    seq
    .............................................................................aaCgaacuuuuuggccaau................    1    1    seq
```
